# Prevalence and clinical features of severe diabetic ketoacidosis treated in pediatric intensive care unit: a 5-year monocentric experience

**DOI:** 10.1186/s13052-023-01448-1

**Published:** 2023-05-20

**Authors:** Stefano Passanisi, Giuseppina Salzano, Pietro Basile, Bruno Bombaci, Flavia Caime, Immacolata Rulli, Mariella Valenzise, Eloisa Gitto, Fortunato Lombardo

**Affiliations:** 1 Department of Human Pathology in Adult and Developmental Age “Gaetano Barresi”, University of Messina, Via Consolare Valeria 1, 98124 Messina, ME Italy; 2 Department of Human Pathology in Adult and Developmental Age “Gaetano Barresi”, Neonatal and Pediatric Intensive Care Unit, University of Messina, Messina, Italy

**Keywords:** Acute kidney injury, Bicarbonate, Cerebral edema, Children, Deep vein thrombosis, Glasgow Coma Scale, Immigrant background, Type 1 diabetes

## Abstract

**Background:**

Diabetic ketoacidosis (DKA) is one of the most alarming concerns in the management of type 1 diabetes (T1D) in pediatric age. Prevalence of DKA at the onset of diabetes ranges from 30 to 40%. In selected cases of severe DKA, admission to pediatric intensive care unit (PICU) should be considered.

**Methods:**

This study aims to assess the prevalence of severe DKA treated in PICU in our 5-year monocentric experience. Secondary outcome of the study was to describe the main demographical and clinical features of individuals who required admission to PICU. All clinical data were collected by retrospectively reviewing the electronic medical records of children and adolescents with diabetes hospitalized in our University Hospital from January 2017 to December 2022.

**Results:**

During the study period, 103 children and adolescents were newly diagnosed with T1D. Among these, 51.5% presented clinical criteria for DKA and almost 10% needed to be treated in PICU. A higher rate of new T1D diagnoses was observed in 2021, as well as episodes of severe DKA being more frequent than in previous years. Due to severe clinical manifestations of DKA, 10 subjects (9.7%) with T1D onset needed to be treated in PICU. Of these, four children were younger than 5. The great majority came from a low household income and some of them had also immigrant background. The most common complication of DKA was acute kidney injury presented by four children. Other complications were cerebral edema, papilledema and acute esophageal necrosis. A 15-year-old girl had deep vein thrombosis (DVT) that evolved into multiple organ failure leading to death.

**Conclusions:**

Our findings demonstrated that severe DKA is still quite common in children and adolescents at T1D onset, especially in some areas such as Southern Italy. Public awareness campaigns should be increasingly promoted to facilitate the recognition of early symptoms of diabetes and to reduce morbidity and mortality related to DKA.

## Background

Diabetic ketoacidosis (DKA) is the major cause of morbidity and mortality in children with type 1 diabetes (T1D). It can occur in previously undiagnosed individuals with diabetes or may be secondary to omission of insulin injections, insulin pump failure, or inadequate management of an infection. Prevalence of DKA at the onset of T1D ranges from 30 to 40% in high-income countries [1, 2]. DKA is caused by absolute or relative insulin deficiency and high counterregulatory hormone concentrations, which accelerate catabolic state with increased glucose production via glycogenolysis and gluconeogenesis and impaired peripheral glucose utilization, resulting in hyperglycemia and hyperosmolality. Insulin deficiency and increased counterregulatory hormone levels also cause lipolysis and accumulation of ketones leading to ketonemia and metabolic acidosis [3]. According to the consensus statement from the International Society for Pediatric and Adolescent Diabetes (ISPAD) [4], DKA is defined by the presence of the following biochemical signs: blood sugar > 200 mg/dl (11 mmol/L), venous pH < 7.3 or serum bicarbonate < 18 mmol/L, beta-hydroxybutyrate > 3 mmol/L or moderate—large urine ketones. The severity of DKA is determined by the degree of acidosis: mild (pH < 7.3 or serum bicarbonate < 18 mmol/L), moderate (pH < 7.2 serum bicarbonate < 10 mmol/L), severe (pH 7.1, serum bicarbonate < 5 mmol/L). DKA therapy aims at correcting acidosis, hyperketonemia, dehydration, and dyselectrolytemia if present, along with a gradual normalization of serum glucose values. Mortality rates for DKA in Western countries range from 0.15 to 0.31% [5]. The most insidious complication of DKA is cerebral edema (CE), which occurs in 0.3—1% of cases and is fatal in 20—30% of cases [6]. Admission to pediatric intensive care unit (PICU) should be considered for individuals with several risk factors for CE (i.e. pre-school age, severe acidosis, elevated blood urea nitrogen, low partial carbon dioxide pressure), and for all those with severe DKA characterized by depressed level of consciousness, impaired circulation, and long duration of symptoms [4].

## Methods

Aim of this retrospective study was to assess the prevalence of severe DKA treated in PICU in our monocentric experience both among new diagnosis of T1D and among secondary DKA cases. Secondary outcome of the study was to describe the main demographical and clinical features of individuals who required admission to PICU. We selected children and adolescents admitted to our tertiary-care center between January 2017 and December 2021 with a new diagnosis of T1D. Our University Hospital, set in the metropolitan city of Messina, includes both a regional reference center for pediatric diabetes and a PICU. Children and adolescents up to 16 years with newly diagnosed T1D from eastern Sicily and from the nearby province of Reggio Calabria, in the Calabria region, are regularly admitted to our facility. Our territorial prevention strategies for DKA include the identification of subjects at risk based on the presence of a first-degree relative with known T1D and public awareness campaigns in schools. The study was conducted in compliance with the Helsinki Declaration, good clinical practice and all applicable laws and regulations. Our investigation was not subject to ethical committee approval since it was limited to anonymized and unidentifiable data routinely collected in our clinical practice. The diagnosis of DKA was made according to the latest ISPAD Clinical Practice Consensus Guidelines [7].

Demographic and socioeconomic information including gender, age, family history of T1D, immigration background, family income, and clinical data such as anthropometric parameters, venous pH, serum bicarbonate levels, glycated hemoglobin (HbA1c) values, diabetes-specific autoantibody profiles, and clinical outcomes were collected. Regarding individuals admitted to PICU with severe DKA, further biochemical data including blood glucose, ketonemia, blood urea nitrogen (BUN), serum creatinine, serum potassium, and c-peptide levels were considered. Clinical features at the time of PICU admission, type of DKA treatment, and eventual occurrence of complications were also recorded. Cases of acute kidney injury (AKI) were defined according to Kidney Disease Improving Global Outcomes guidelines by an increase of at least 1.5 times the estimated baseline of serum creatinine [8].

Descriptive statistics were performed, and results are expressed using frequencies and percentages for qualitative variables, median and interquartile ranges for quantitative variables. A post-hoc subgroup analysis was made to compare clinical data between different subgroups of individuals newly diagnosed with T1D according to the severity of DKA at the onset of disease. ANOVA test was applied for quantitative parameters and χ2 test or Fisher’s exact test, when appropriate, was used for categorical variables. Statistical significance level was set at *p *< 0.05.

## Results

**Fig. 1:**
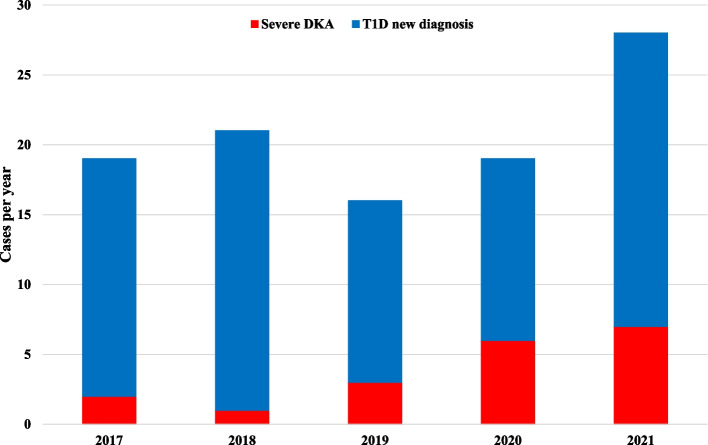
New cases of T1D and hospitalizations for severe DKA over the period 2017–2021

**Fig. 2:**
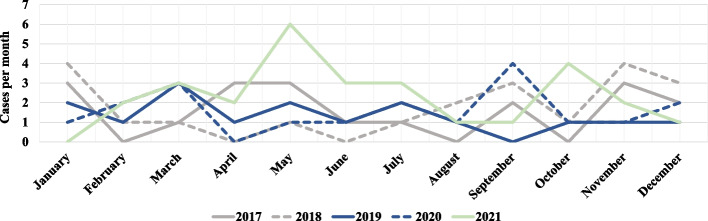
Seasonality of new cases of T1D over the study period

During the study period, 103 children and adolescents (62.1% males) with median age 9.5 years (6.5; 12.3) were newly diagnosed with T1D. Among these, 53 (51.5%) presented clinical criteria for DKA, of which 19 (18.4%) had severe DKA. A higher rate of new T1D diagnoses, especially during warm months, was observed in 2021, as well as cases of severe DKA being more frequent than in previous years (Figs. 1 and  2). Demographic, socioeconomic, clinical characteristics of subjects with T1D onset, and comparison between groups according the severity of DKA are summarized in Table 1.

**Table 1:** Clinical features of new cases of type 1 diabetes during the study period and comparison between subgroups according to the severity of diabetic ketoacidosis

	**No DKA**	**Mild DKA**	**Moderate DKA**	**Severe DKA**	***p* value**
**Number of patients**	50 (48.5%)	25 (24.3%)	9 (8.7%)	19 (18.4%)	
**Age (years)**	10.3 (6.7; 12.3)	9.1 (7.2; 12.1)	9.8 (8.7; 13)	6.0 (1.9; 11.5)	0.019*
**Gender**					0.122
Male	30 (60%)	18 (72%)	6 (66.7%)	10 (52.6%)	
Female	20 (40%)	7 (28%)	3 (33.3%)	9 (47.4%)	
**Height (Z-score)**	0.49 (-0.72; 0.90)	-0.03 (-0.39; 0.81)	-0.56 (-1.08; -0.28)	-0.49 (-0.92; 0.55)	0.374
**Weight (Z-score)**	-0.06 (-0.59; 0.81)	-0.35 (-1.05; 1.11)	-0.04 (-0.58; 0.1)	-0.26 (-0.92; 0.77)	0.827
**BMI (Z-score)**	0.52 (-0.44; 1.62)	0.36 (-0.77; 1.96)	0.51 (-0.06; 1.00)	-0.14 (-0.82; 0.95)	0.465
**Family history of T1D (1**^**st**^** degree)**	6 (12%)	2 (8%)	1 (11.1%)	0	n.a
**Low household income**	23 (46%)	12 (48%)	4 (44.4%)	11 (57.9%)	0.279
**Immigrant background**	3 (6%)	0	0	5 (26.3%)	n.a
**Venous pH**	7.35 (7.33; 7.38)	7.27 (7.24; 7.29)	7.14 (7.14; 7.2)	6.91 (6.85; 7.00)	< 0.001*
**Serum bicarbonate (mmol/L)**	21.4 (20.1; 22.9)	16.2 (14.7; 18.2)	10.7 (10.6; 11.1)	6.5 (5.1; 7.7)	< 0.001*
**HbA1c at onset (%)**	11.2 (8.9; 13.7)	11.9 (9.6; 13.4)	12.4 (11.3; 14)	11.1 (10.7; 12.6)	0.874
**T1D autoantibodies**	35 (70%)	21 (84%)	6 (66.7%)	14 (73.7%)	0.741
**Cerebral edema**	0	0	0	1 (5.3%)	n.a
**Mortality**	0	0	0	1 (5.3%)	n.a

*BMI* Body mass index, *DKA* Diabetic ketoacidosis, *HbA1c* Glycated hemoglobin, *n.a* Not available *T1D* Type 1 diabetes

^*^significant *p-*value

### Patients admitted to PICU

Due to severe clinical manifestations of DKA, 10 individuals (9.7%) with T1D onset needed to be treated in PICU. Their demographic and clinical features are shown in Table 2. Seven of them (70%) were male, and age ranged from 1.0 to 14.9 years. Four children were younger than 5. The majority came from a low household income (< €15.000), and almost half of them (40%) came from an immigrant background. None of them had a first-degree relative with T1D. Median blood glucose at admission was 525 mg/dl (449; 710), while HbA1c was 10.9% (10.2; 12.6). Basal c-peptide levels were < 0.8 ng/mL in most individuals, and T1D-specific autoimmunity was absent in only one child. Polyuria, polydipsia, Kussmaul’s breathing and gastrointestinal symptoms (i.e. abdominal pain, vomiting) were equally distributed among individuals as presenting symptoms. Half of the subjects presented with severe neurological impairment (Glasgow coma scale < 9), while four (40%) had a Glasgow coma scale between 9 and 13. At PICU admission, median venous pH was 6.88 (6.84; 6.94), serum bicarbonate level was 5 mmol/L (3.3; 5.7), and ketonemia was 5.5 mmol/L (4.5; 6.3). Abnormal levels of potassium before the start of DKA correction were found in only one child, who presented hyperkalemia. Fluid replacement and intravenous insulin administration was practiced for a median period of 40.5 h (31.5; 66), while bicarbonate therapy was required in three cases. The most common complication of DKA was AKI presented by four individuals. Signs of papilledema were found in one boy and an overt condition of CE was diagnosed in another subject. A 1-year-old child experienced black esophagus. Finally, a 15-year-old girl had deep vein thrombosis (DVT) that evolved to multiple organ failure resulting in death.

**Table 2:** Summary of demographic, clinical and biochemical data of patients newly diagnosed with type 1 diabetes who required admission to pediatric intensive care unit due to severe diabetic ketoacidosis. Qualitative data are expressed by using frequencies and percentages, numerical data are indicated as median and interquartile ranges

Number of patients	10
Age (years)	5.6 (2.7; 8)
**Gender**	
Male	7 (70%)
Female	3 (30%)
Height (Z-score)	0.34 (-0.83; 1.34)
Weight (Z-score)	0.82 (-0.15; 0.93)
BMI (Z-score)	0.24 (-0.32; 1.10)
Family history of T1D (1^st^ degree)	0
Low household income	8 (80%)
Immigrant background	4 (40%)
**Clinical presentation**	
Kussmaul’s breathing	7 (70%)
Polyuria and polydipsia	7 (70%)
Gastrointestinal symptoms	7 (70%)
Severe neurological impairment (GCS < 9)	5 (50%)
Moderate neurological impairment (GCS between 9 and 13)	4 (40%)
**Biochemical data**	
Blood glucose at admission (mg/dl)	525 (449; 710)
Venous pH	6.88 (6.84; 6.94)
Serum bicarbonate (mmol/L)	5 (3.3; 5.7)
Base excess (mmol/L)	-28.4 (-30.2; -25.6)
Ketonemia (mmol/L)	5.5 (4.5; 6.3)
BUN (mg/dl)	27.5 (23; 34.2)
Serum creatinine (mg/dl)	0.6 (0.6; 0.9)
Serum potassium (mg/dl)	3.9 (3.4; 4.7)
HbA1c at onset (%)	10.9 (10.2; 12.6)
Autoantibodies	9 (90%)
Basal C-peptide (ng/ml)	0.26 (0.05; 0.4)
**DKA treatment**	
Duration of DKA treatment (hours)	40.5 (31.5; 66)
Bicarbonate administration	3 (30%)
**Outcomes**	
Acute kidney injury	4 (40%)
Cerebral edema	1 (10%)
MOF	1 (10%)
Other complications	1 (10%)
Mortality	1 (10%)

*BMI* Body mass index, *BUN* Blood urea nitrogen, *DKA* Diabetic ketoacidosis, *GCS* Glasgow coma scale, *HbA1c* Glycated hemoglobin, *MOF* Multiple organ failure, *PICU* Pediatric intensive care unit, *T1D* Type 1 diabetes

### Secondary DKA

Additionally, 7 individuals already diagnosed with T1D presented were hospitalized due to secondary DKA during the entire study period. Of these, two (28.5%) needed to be treated in PICU. Both subjects came from an immigrant and low household income background. In the first case of an adolescent female on multiple daily injection insulin therapy, secondary DKA was caused by bad therapeutic management characterized by recurrent omission of insulin administrations for several days. In the other case, a 6-year-old female child was forced to discontinue insulin therapy for a long time due to adverse circumstances related to a clandestine migration journey. DKA was complicated by the occurrence of AKI, CE, and multiple organ failure but without chronic sequelae (Table 3).

**Table 3:** Demographic, clinical and biochemical data of two patients with already diagnosed type 1 diabetes admitted to pediatric intensive care unit for severe secondary diabetic ketoacidosis

	**Patient 1**	**Patient 2**
Age (years)	13.3	6.5
Gender	Female	Female
Height (Z-score)	-0.75	-1.46
Weight (Z-score)	-2.10	-2.30
BMI (Z-score)	-1.96	-1.94
Family history of T1D (1^st^ degree)	No	Unknown
Low household income	Yes	Yes
Immigrant background	Yes	Yes
**Clinical presentation**		
Kussmaul’s breathing	Yes	Yes
Polyuria and polydipsia	Yes	No
Gastrointestinal symptoms	Yes	No
Severe neurological impairment (GCS < 9)	No	Yes
Moderate neurological impairment (GCS between 9 and 13)	No	No
**Biochemical data**		
Blood glucose at admission (mg/dl)	750	698
Venous pH	6.80	6.83
Serum bicarbonate (mmol/L)	5.6	3.0
Base excess (mmol/L)	-31	Unknown
BUN (mg/dl)	74	109
Serum creatinine (mg/dl)	1.3	1.2
Serum potassium (mg/dl)	6.2	3.4
HbA1c (%)	8.1	7.6
**DKA treatment**		
Duration of DKA treatment (hours)	96	48
Bicarbonate administration	No	No
**Outcomes**		
Acute kidney injury	No	Yes
Cerebral edema	No	Yes
MOF	No	Yes
Mortality	No	No

*BMI* Body mass index, *BUN* Blood urea nitrogen, *DKA* Diabetic ketoacidosis, *GCS* Glasgow coma scale, *HbA1c* Glycated hemoglobin, *MOF* Multiple organ failure, *PICU* Pediatric intensive care unit, *T1D* Type 1 diabetes

## Discussion

Our single-center 5-year experience revealed that prevalence of DKA at T1D onset was 51.5% and almost 10% of young individuals newly diagnosed with T1D needed to be treated in PICU. Despite numerous public health campaigns being promoted in several countries to increase awareness and knowledge about pediatric diabetes during the past years [9–11], initial symptoms of T1D are often overlooked, and DKA is still common among children and adolescents [12]. However, frequency of DKA greatly varies between different areas, with lower rates in areas such as the Sardinia region, Northern Europe and Canada, where populations are likely more sensitized about the risk of T1D due to high incidence rates of the disease [13].

Interestingly, prevalence of severe DKA in our center was relatively higher in comparison to other Italian studies [14] and to other developed countries [15–17], but in accordance with recent epidemiologic data from Southern Italy [18, 19]. Our analysis also showed an apparent increase in severity of DKA during the period 2020–2021. This trend may be consistent with other studies [20–23], which attribute delays of T1D diagnosis and subsequent more severe presentation to indirect effects of the COVID-19 pandemic, such as the interruption of some outpatient services or omission of medical consultations due to fear of contracting SARS-CoV-2 infection by families. In accordance with our experience, a modification of the seasonality of T1D incidence rate during the pandemic years, with unprecedented peaks during warm months, has been observed in several countries [24]. This finding can be attributable to social distancing measures that may have altered the diffusion of epidemic viruses known to act as environmental triggers for the development of T1D. A direct effect of SARS-CoV-2 against β-cells through the binding to ACE2 receptors expressed in pancreatic tissue is currently under investigation [25].

Few studies reported data on rates of hospitalizations in PICU at T1D onset. Our prevalence of PICU admission due to severe DKA is lower than that found by a recent single-center study from Croatia [26]. Differences between rates of DKA-related hospitalizations in pediatric critical care areas may be related to disparities in territorial availability of PICU or to different levels of expertise on DKA management in pediatric non-intensive units.

Severity of DKA presentation may be influenced by several factors, including those related to socioeconomic background of subjects. Our data showed that a consistent number of children belonged to low socioeconomic status had severe DKA or required admission to PICU as already demonstrated by larger scale studies [14, 27]. Our findings revealed that also immigrant background and ethnic minority status have been associated to higher likelihood of developing severe DKA at T1D onset, as a result of language and cultural barriers that may hamper the awareness of the disease and delay the access to healthcare structures [14, 28, 29].

Our results confirmed that pre-school age remains a notable risk factor for developing severe DKA [18, 28]. The main hypothesis to explain the more severe metabolic decompensation in younger children is that the process of pancreatic beta cell destruction seems to be faster and more aggressive due to the activation of the pro-inflammatory cytokine storm [30, 31]. In addition, early symptoms of hyperglycemia are usually poorly recognized in this age group [32].

The use of bicarbonate to counteract the state of acidosis in children and adolescents with DKA has been widely discouraged by clinical evidence [33], due to the increased risk of harmful complications such as CE and hypokalemia. However, international DKA guidelines suggest the administration of bicarbonate in selected cases, including severe hyperkalemia or extreme acidosis that may compromise cardiac function [4]. In our experience, the use of sodium bicarbonate was restricted to those subjects with persistence of severe acidosis after more than 48 h of DKA correction, despite the normalization of blood ketones and glucose levels.

AKI is one of the most frequently encountered ongoing complications of severe DKA and is generally related to decreased renal perfusion caused by intravascular volume depletion [34]. In our experience, AKI was found in 40% individuals admitted in PICU and in 8.9% of total DKA cases. These findings are in line with data previously reported by other studies [35–37]. No evidence of further sequelae during the follow-up was found in our records. CE is the most alarming complication of DKA and the leading cause of mortality [38]. Some authors have hypothesized that cerebral hypoperfusion and reperfusion effects associated with neuroinflammation play a key role in the occurrence of CE [6, 39, 40]. Prolonged duration of DKA, severity of dehydration and acidosis, persistent hyponatremia, hypocapnia and bicarbonate therapy are factors that may facilitate the development of CE [41–43]. About 20–30% of CE survivors have residual disabilities ranging from mild neurological impairment to a vegetative state [44, 45]. In our study, only one case of cerebral edema was detected without any neurological sequelae.

In our study cohort, an interesting case of acute esophageal necrosis (AEN), also known as Gurvits syndrome or black esophagus, was present. AEN is an extremely rare condition characterized by necrotic lesions of esophageal mucosal and submucosal layers as the result of a combination of tissue hypoperfusion, impaired local defense barriers and massive influx of gastric contents [46–48]. Gastrointestinal bleeding is the most frequent clinical manifestation, often associated with early signs of hemodynamic instability [49].

Overall mortality in children with DKA ranges from 0.15 to 0.35% in Western countries such as the UK, Canada, USA and increases to 3.4—13.4% in low-middle income countries [5, 50]. In our study, only one individual died. The cause of death was multi organ failure secondary to DVT, which is a rare complication in children and adolescents. Shock and dehydration are the main causes for the state of hypercoagulability, which is the trigger of the coagulative cascade and venous stasis. The presence of femoral venous cannulation increases the risk of developing DVT [51].

The occurrence of DKA in subjects with already known T1D represents a major concern in clinical practice. In our experience, it was often characterized by severe presentation and required hospitalization in PICU in almost one third of cases. The most common causes of recurrent DKA are accidental or deliberate insulin omission, inadequate management of sick days, and unnoticed insulin pump failure. Support of clinical psychologists and a proper therapeutic education are crucial in the prevention of these events [4].

## Conclusions

Severe DKA is still quite common in children and adolescents at T1D onset, especially in some areas such as Southern Italy. In a consistent number of cases, individuals with severe DKA need PICU treatment for the occurrence of life-threatening clinical manifestations, including impairment of vital functions and multiple organ failure. Our study highlights the need to increasingly promote public awareness campaigns to facilitate the recognition of early symptoms of diabetes and to reduce morbidity and mortality related to DKA.

## Data Availability

The datasets used and/or analyzed during the current study are available from the corresponding author on reasonable request.

## Abbreviations

AEN: Acute esophageal necrosis
AKI: Acute kidney injury
CE: Cerebral edema
DKA: Diabetic ketoacidosis
DVT: Deep vein thrombosis
HbA1c: Glycated hemoglobin
ISPAD: International Society for Pediatric and Adolescent Diabetes
PICU: Pediatric intensive care unit
T1D: Type 1 diabetes

## References

[1] Jensen ET, Stafford JM, Saydah S, D’Agostino RB, Dolan LM, Lawrence JM (2021). Increase in Prevalence of Diabetic Ketoacidosis at Diagnosis Among Youth With Type 1 Diabetes: The SEARCH for Diabetes in Youth Study. Diabetes Care.

[2] Kao KT, Islam N, Fox DA, Amed S (2020). Incidence Trends of Diabetic Ketoacidosis in Children and Adolescents with Type 1 Diabetes in British Columbia. Canada J Pediatr.

[3] Lapolla A, Amaro F, Bruttomesso D, Di Bartolo P, Grassi G, Maffeis C (2020). Diabetic ketoacidosis: A consensus statement of the Italian Association of Medical Diabetologists (AMD), Italian Society of Diabetology (SID), Italian Society of Endocrinology and Pediatric Diabetoloy (SIEDP). Nutr Metab Cardiovasc Dis.

[4] Glaser N, Fritsch M, Priyambada L, Rewers A, Cherubini V, Estrada S (2022). ISPAD clinical practice consensus guidelines 2022: Diabetic ketoacidosis and hyperglycemic hyperosmolar state. Pediatr Diabetes.

[5] Curtis JR, To T, Muirhead S, Cummings E, Daneman D (2002). Recent trends in hospitalization for diabetic ketoacidosis in ontario children. Diabetes Care.

[6] Azova S, Rapaport R, Wolfsdorf J (2021). Brain injury in children with diabetic ketoacidosis: Review of the literature and a proposed pathophysiologic pathway for the development of cerebral edema. Pediatr Diabetes.

[7] Libman I, Haynes A, Lyons S, Pradeep P, Rwagasor E, Tung JY (2022). ISPAD Clinical Practice Consensus Guidelines 2022: Definition, epidemiology, and classification of diabetes in children and adolescents. Pediatr Diabetes.

[8] Khwaja A (2012). KDIGO clinical practice guidelines for acute kidney injury. Nephron Clin Pract.

[9] Vanelli M, Chiari G, Ghizzoni L, Costi G, Giacalone T, Chiarelli F (1999). Effectiveness of a prevention program for diabetic ketoacidosis in children. An 8-year study in schools and private practices. Diabetes Care..

[10] Derraik JGB, Cutfield WS, Maessen SE, Hofman PL, Kenealy T, Gunn AJ (2018). A brief campaign to prevent diabetic ketoacidosis in children newly diagnosed with type 1 diabetes mellitus: The NO-DKA Study. Pediatr Diabetes.

[11] King BR, Howard NJ, Verge CF, Jack MM, Govind N, Jameson K (2012). A diabetes awareness campaign prevents diabetic ketoacidosis in children at their initial presentation with type 1 diabetes. Pediatr Diabetes.

[12] Rabbone I, Maltoni G, Tinti D, Zucchini S, Cherubini V, Bonfanti R (2020). Diabetic ketoacidosis at the onset of disease during a national awareness campaign: a 2-year observational study in children aged 0–18 years. Arch Dis Child.

[13] Usher-Smith JA, Thompson M, Ercole A, Walter FM (2012). Variation between countries in the frequency of diabetic ketoacidosis at first presentation of type 1 diabetes in children: a systematic review. Diabetologia.

[14] Cherubini V, Marino M, Scaramuzza AE, Tiberi V, Bobbio A, Delvecchio M (2022). The Silent Epidemic of Diabetic Ketoacidosis at Diagnosis of Type 1 Diabetes in Children and Adolescents in Italy During the COVID-19 Pandemic in 2020. Front Endocrinol.

[15] Choleau C, Maitre J, Filipovic Pierucci A, Elie C, Barat P, Bertrand AM (2014). Ketoacidosis at diagnosis of type 1 diabetes in French children and adolescents. Diabetes Metab.

[16] Neu A, Hofer SE, Karges B, Oeverink R, Rosenbauer J, Holl RW (2009). Ketoacidosis at diabetes onset is still frequent in children and adolescents: a multicenter analysis of 14,664 patients from 106 institutions. Diabetes Care.

[17] Hekkala A, Knip M, Veijola R (2007). Ketoacidosis at diagnosis of type 1 diabetes in children in northern Finland: temporal changes over 20 years. Diabetes Care.

[18] Cherubini V, Skrami E, Ferrito L, Zucchini S, Scaramuzza A, Bonfanti R (2016). High frequency of diabetic ketoacidosis at diagnosis of type 1 diabetes in Italian children: a nationwide longitudinal study, 2004–2013. Sci Rep.

[19] Passanisi S, Salzano G, Aloe M, Bombaci B, Citriniti F, De Berardinis F (2022). Increasing trend of type 1 diabetes incidence in the pediatric population of the Calabria region in 2019–2021. Ital J Pediatr.

[20] McGlacken-Byrne SM, Drew SEV, Turner K, Peters C, Amin R (2021). The SARS-CoV-2 pandemic is associated with increased severity of presentation of childhood onset type 1 diabetes mellitus: A multi-centre study of the first COVID-19 wave. Diabet Med J.

[21] Rabbone I, Schiaffini R, Cherubini V, Maffeis C, Scaramuzza A, Diabetes Study Group of the Italian Society for Pediatric Endocrinology and Diabetes (2020). Has COVID-19 Delayed the Diagnosis and Worsened the Presentation of Type 1 Diabetes in Children?. Diabetes Care..

[22] Qeadan F, Tingey B, Egbert J, Pezzolesi MG, Burge MR, Peterson KA (2022). The associations between COVID-19 diagnosis, type 1 diabetes, and the risk of diabetic ketoacidosis: A nationwide cohort from the US using the Cerner Real-World Data. PLoS One.

[23] Birkebaek NH, Kamrath C, Grimsmann JM, Aakesson K, Cherubini V, Dovc K, et al. Impact of the COVID-19 pandemic on long-term trends in the prevalence of diabetic ketoacidosis at diagnosis of paediatric type 1 diabetes: an international multicentre study based on data from 13 national diabetes registries. Lancet Diabetes Endocrinol. 2022;S2213–8587(22):00246–7. 10.1016/S2213-8587(22)00246-7 PMC9597608 36202118

[24] Reschke F, Lanzinger S, Herczeg V, Prahalad P, Schiaffini R, Mul D (2022). The COVID-19 Pandemic Affects Seasonality, With Increasing Cases of New-Onset Type 1 Diabetes in Children, From the Worldwide SWEET Registry. Diabetes Care.

[25] Wu CT, Lidsky PV, Xiao Y, Lee IT, Cheng R, Nakayama T (2021). SARS-CoV-2 infects human pancreatic β cells and elicits β cell impairment. Cell Metab.

[26] Lah Tomulić K, Matko L, Verbić A, Milardović A, Severinski S, Kolić I (2022). Epidemiologic Characteristics of Children with Diabetic Ketoacidosis Treated in a Pediatric Intensive Care Unit in a 10-Year-Period: Single Centre Experience in Croatia. Med Kaunas Lith.

[27] Gesuita R, Maffeis C, Bonfanti R, Cardella F, Citriniti F, D’Annunzio G (2020). Socioeconomic Inequalities Increase the Probability of Ketoacidosis at Diagnosis of Type 1 Diabetes: A 2014–2016 Nationwide Study of 2,679 Italian Children. Front Pediatr.

[28] Cherubini V, Grimsmann JM, Åkesson K, Birkebæk NH, Cinek O, Dovč K (2020). Temporal trends in diabetic ketoacidosis at diagnosis of paediatric type 1 diabetes between 2006 and 2016: results from 13 countries in three continents. Diabetologia.

[29] Duca LM, Reboussin BA, Pihoker C, Imperatore G, Saydah S, Mayer-Davis E (2019). Diabetic ketoacidosis at diagnosis of type 1 diabetes and glycemic control over time: The SEARCH for diabetes in youth study. Pediatr Diabetes.

[30] Atkinson MA, von Herrath M, Powers AC, Clare-Salzler M (2015). Current concepts on the pathogenesis of type 1 diabetes–considerations for attempts to prevent and reverse the disease. Diabetes Care.

[31] Passanisi S, Salzano G, Gasbarro A, Urzì Brancati V, Mondio M, Pajno GB (2020). Influence of Age on Partial Clinical Remission among Children with Newly Diagnosed Type 1 Diabetes. Int J Environ Res Public Health.

[32] Bizzarri C, Benevento D, Ciampalini P, Patera Ippolita P, Schiaffini R, Migliaccio A (2010). Clinical presentation and autoimmune characteristics of very young children at the onset of type 1 diabetes mellitus. J Pediatr Endocrinol Metab.

[33] Green SM, Rothrock SG, Ho JD, Gallant RD, Borger R, Thomas TL (1998). Failure of adjunctive bicarbonate to improve outcome in severe pediatric diabetic ketoacidosis. Ann Emerg Med.

[34] Huang JX, Casper TC, Pitts C, Myers S, Loomba L, Ramesh J (2022). Association of Acute Kidney Injury During Diabetic Ketoacidosis With Risk of Microalbuminuria in Children With Type 1 Diabetes. JAMA Pediatr.

[35] Myers SR, Glaser NS, Trainor JL, Nigrovic LE, Garro A, Tzimenatos L (2020). Frequency and Risk Factors of Acute Kidney Injury During Diabetic Ketoacidosis in Children and Association With Neurocognitive Outcomes. JAMA Netw Open.

[36] Chen J, Zeng H, Ouyang X, Zhu M, Huang Q, Yu W (2020). The incidence, risk factors, and long-term outcomes of acute kidney injury in hospitalized diabetic ketoacidosis patients. BMC Nephrol.

[37] Galindo RJ, Pasquel FJ, Vellanki P, Zambrano C, Albury B, Perez-Guzman C (2021). Biochemical Parameters of Diabetes Ketoacidosis in Patients with End-stage Kidney Disease and Preserved Renal Function. J Clin Endocrinol Metab.

[38] Levitsky LL (2004). Symptomatic cerebral edema in diabetic ketoacidosis: the mechanism is clarified but still far from clear. J Pediatr.

[39] Glaser NS, Wootton-Gorges SL, Marcin JP, Buonocore MH, Dicarlo J, Neely EK (2004). Mechanism of cerebral edema in children with diabetic ketoacidosis. J Pediatr.

[40] Glaser N, Yuen N, Anderson SE, Tancredi DJ, O’Donnell ME (2010). Cerebral metabolic alterations in rats with diabetic ketoacidosis: effects of treatment with insulin and intravenous fluids and effects of bumetanide. Diabetes.

[41] Agarwal N, Dave C, Patel R, Shukla R, Kapoor R, Bajpai A (2020). Factors Associated With Cerebral Edema at Admission in Indian Children with Diabetic Ketoacidosis. Indian Pediatr.

[42] Raghunathan V, Jevalikar G, Dhaliwal M, Singh D, Sethi SK, Kaur P (2021). Risk Factors for Cerebral Edema and Acute Kidney Injury in Children with Diabetic Ketoacidosis. Indian J Crit Care Med Peer-Rev.

[43] Jeziorny K, Waszczykowska A, Barańska D, Szadkowska A, Młynarski W, Zmysłowska A (2020). Can we effectively predict the occurrence of cerebral edema in children with ketoacidosis in the course of type 1 diabetes? - case report and literature review. J Pediatr Endocrinol Metab.

[44] Cameron FJ, Scratch SE, Nadebaum C, Northam EA, Koves I, Jennings J (2014). Neurological consequences of diabetic ketoacidosis at initial presentation of type 1 diabetes in a prospective cohort study of children. Diabetes Care.

[45] Glaser N, Barnett P, McCaslin I, Nelson D, Trainor J, Louie J (2001). Risk factors for cerebral edema in children with diabetic ketoacidosis. The Pediatric Emergency Medicine Collaborative Research Committee of the American Academy of Pediatrics. N Engl J Med..

[46] Gurvits GE, Shapsis A, Lau N, Gualtieri N, Robilotti JG (2007). Acute esophageal necrosis: a rare syndrome. J Gastroenterol.

[47] Kitawaki D, Nishida A, Sakai K, Owaki Y, Nishino K, Noda Y (2022). Gurvits syndrome: a case of acute esophageal necrosis associated with diabetic ketoacidosis. BMC Gastroenterol.

[48] Quitadamo P, Caruso F, Bucci C, Del Monaco C, Verde A, Zanfardino A (2021). Black oesophagus in an adolescent with type 2 diabetes. Lancet Diabetes Endocrinol.

[49] Choksi V, Dave K, Cantave R, Shaharyar S, Joseph J, Shankar U (2017). «Black Esophagus» or Gurvits Syndrome: A Rare Complication of Diabetic Ketoacidosis. Case Rep Gastrointest Med.

[50] Poovazhagi V (2014). Risk factors for mortality in children with diabetic ketoacidosis from developing countries. World J Diabetes.

[51] Davis J, Surendran T, Thompson S, Corkey C (2007). DKA, CVL and DVT. Increased risk of deep venous thrombosis in children with diabetic ketoacidosis and femoral central venous lines. Ir Med J..

